# Short and long-term effectiveness of external shock wave therapy for chronic pelvic pain syndrome in men

**DOI:** 10.1080/2090598X.2023.2207415

**Published:** 2023-05-14

**Authors:** Kareim Khalafalla, Ahmed Albakr, Walid El Ansari, Ahmad Majzoub, Haitham Elbardisi, Khalid AlRumaihi, Mohamed Arafa

**Affiliations:** a Department of Urology, Hamad General Hospital, Doha, Qatar; b Division of Urology, Department of Surgery, University of Texas McGovern Medical School, Houston, TX, USA; c Department of Urology, MD Anderson Cancer Center, Houston, TX, USA; d Department of Surgery, Hamad Medical Corporation, Doha, Qatar; e Department of Population Health, College of Medicine, Qatar University, Doha, Qatar; f Institute for Population Health, Weill Cornell Medicine – Qatar, Doha, Qatar; g Department of Urology, Weill Cornell Medicine – Qatar, Doha, Qatar; h Department of Urology, Qatar University, Doha, Qatar; i Department of Andrology, Cairo University, Cairo, Egypt

**Keywords:** Chronic pelvic pain syndrome, External shock wave therapy (ESWT), NIH-chronic prostatitis symptom index

## Abstract

**Introduction:**

Chronic pelvic pain syndrome (CPPS) is a frequent urological diagnosis that affects men’s quality of life. Extracorporeal shockwave therapy (ESWT) is a recent treatment option for patients with CPPS. We evaluated ESWT’s short and long – term efficacy in managing CPPS.

**Methods:**

This prospective self-controlled study included 75 patients diagnosed with CPPS at our tertiary pelvic pain clinic between January 2017-June 2019. Patients were referred for ESWT and received four sessions one week apart. The National Institute for Health – Chronic Prostatitis Symptom Index (NIH – CPSI) questionnaire was used to assess patients’ symptom severity before starting therapy and at 0, 12 and 26 weeks after completing ESWT. Demographics, clinical data and complications were also recorded.

**Results:**

Patients’ mean age was 37.9 ± 8.6 years, and mean duration of symptoms was 5 ± 4.5 years. Compared to pre-treatment scores, all patients exhibited improvements across all NIH – CPSI domains directly after completing ESWT (week 0 post-treatment), with a mean difference improvement of 9.26 ± 5.7, 5.2 ± 3.4, 1.19 ± 2.18 and 2.88 ± 2.46 points in the total, pain, urinary symptoms, and quality-of-life scores respectively. At 12 weeks after completing ESWT, 80.9% of patients reported improvements, with mean difference improvement of 8.07 ± 7.56, 4.55 ± 4.6, 0.76 ± 2.48, 2.85 ± 2.78 in the total, pain, urinary symptoms, and quality-of-life scores respectively. Again, none of the patients developed any treatment-related complications. At 26 weeks after completing ESWT, 82.4% of patients reported improvements, with mean difference improvement of 8.29 ± 7.7%, 4.92 ± 4.69, 0.75 ± 2.96, 2.5 ± 3.0 in total, pain, urinary symptoms, and quality-of-life scores respectively. None of the patients developed treatment-related complications.

**Conclusions:**

ESWT is a safe and effective treatment modality for patients with CPPS, with short-term improvement in total, pain, urinary symptom, and quality-of-life scores; and long-term improvement in total, pain, and quality-of-life scores.

## Introduction

Chronic prostatitis/chronic pelvic pain syndrome (CP/CPPS) describes one of the common yet most challenging conditions to patients and urologists [1]. The National Institute of Health (NIH) guidelines define CP/CPPS as chronic pelvic pain for at least three out of the six preceding months, often associated with urinary symptoms and sexual dysfunction after exclusion of other identifiable diseases such as neurological bladder diseases, urethral stricture, active urethritis, and urogenital cancer [2]. To date, the exact etiology and pathology of CP/CPPS remain unknown [3].

CP/CPPS is a multifactorial condition affecting men of a wide range of ages and demographics impeding their quality of life [4]. Using variable case-definitions, the prevalence of CP/CPPS in North America, Europe and Asia was estimated at 2–10% [5]. Based on the NIH definition of CP/CPPS, the prevalence of prostatitis-like symptoms in Canada was 9.7% in a survey of 2987 men, and was higher (11.5%) in men<50 years [6]. CP/CPPS prevalence in the Middle East and North Africa (MENA) region remains unknown to date.

In 1999, the International Prostatitis Collaborative Network (IPCN) developed and validated the National Institute of Health chronic prostatitis symptom index (NIH-CPSI) for the diagnosis of CP/CPPS [7]. The index comprises nine items addressing three different aspects of the disease: four items address pain; two assess urinary symptoms; and three appraise quality of life [7]. NIH-CPSI has been widely used globally for the diagnosis, management and follow up of CP/CPPS, and has been translated and validated into many languages, including Arabic [8].

Due to the lack of consensus on the etiology of CP/CPPS, there is no established mono-therapeutic treatment recommendations [3]. Many individual treatment options have been used, including antibiotics, alpha-blockers, immune/neuro-modulatory medications, anti-inflammatory medications, hormonal therapy, and phytotherapy, with varying success rates [3]. In addition, a range of physiotherapeutic modalities have been initially introduced to manage the pain in cases where CP/CPPS is associated with skeletal muscle dysfunction [4]. Such modalities include myofascial physical therapy, acupuncture, sono-electro-magnetic therapy, aerobic exercises, and perineal extracorporeal shock wave therapy (ESWT), and these have been assessed in randomized sham-controlled trials [3]. Nevertheless, the lack of long-term follow-up of therapeutic effect, the small sample sizes and the methodological challenges associated with running large randomized controlled trials using physical therapies hindered the development of solid recommendations for their use as monotherapy [3]. The scope of the current study is on perineal low-energy ESWT.

Generally, focused application of low-energy external shock wave therapy (ESWT) has been employed to manage orthopaedic pain syndromes [9]. ESWT was then expanded to bony tendon attachments, myofascial structures, myofascial pain, as well as in the management of stroke-related upper arm contractures by reducing the passive muscle tone and increasing the range of motion [9]. In 2008, perineal ESWT was applied to the management of CP/CPPS, and its efficacy and safety were assessed [10]. It improved the pain-related symptoms and quality of life of patients, despite that its exact mechanisms of action on soft tissue and nociception were not well understood [10]. Nevertheless, ESWT’s initial potential effects on the multifactorial identity of CP/CPPS showed to be a fruitful field to harvest.

The literature reveals knowledge gaps. Most studies that assessed ESWT suffered from small sample sizes (range 20–30 patients) and/or short-term follow ups (range 2–3 months) [10–15]. Furthermore, the studies that evaluated the longer-term beneficial effects of ESWT reported inconsistent findings, no sustained effect [12] or positive long-term benefits at 6 [13] and 12 months follow up [14,16]. In addition, the literature reveals no consensus on an established ESWT protocol for the management of CP/CPPS in terms of device, dose and duration of shock waves, or number of sessions as well as the tools employed to measure its effects. Likewise, very few studies assessed the effectiveness of ESWT for CP/CPPS among MENA populations [11,16], and some were of short follow up [11], despite anecdotal evidence that CP/CPPS is prevalent across these countries.

Therefore, the current prospective cohort study appraised the short and longer-term effectiveness of ESWT monotherapy among a large sample of CP/CPPS patients in a MENA country (Qatar). We assessed patients referred to our men's chronic pelvic pain clinic at the main tertiary care hospital in Qatar that manages CP/CPPS. Qatar is an Arabian Gulf cosmopolitan country where expatriates comprise about 75% of the population, many from various Middle-Eastern nations. The specific objectives of the study were to assess, among CP/CPPS patients, three domains: pain; urinary symptoms and quality of life; as well as patients’ total NIH-CPSI scores, and compare their pretreatment values with values at 0, 12 and 26 weeks post ESWT. In addition, we undertook a literature review of published articles on ESWT for CP/CPPS and selected examples that illustrated the wide variability in the actual practice/application protocols, as the well as evaluation of its effectiveness.

## Materials and methods

### Setting: Chronic pelvic pain clinic

Men attending the chronic pelvic pain clinic for the first time are thoroughly evaluated with history and physical examination. Different aspects of chronic pelvic pain are assessed and characterized including pain location, description and triggering factors. In addition, pain-related psychosocial symptoms, e.g. stress, anxiety, and depression are appraised. For lower urinary tract symptoms (LUTS) evaluation and bladder and prostate assessment, expressed prostatic secretions and midstream urine samples are cultured. When needed, flexible cystoscopy is performed to exclude anatomical and pathological abnormalities. Depending on the findings, patients are offered pharmacological and/or non-pharmacological therapies, including alpha blockers, antibiotics, antidepressants, anticholinergics, neuroleptics, phytotherapy, physical therapy, mental health therapy and ESWT.

### Study design, ethics and population

Of all the CP/CPPS patients attending our chronic pelvic pain clinic, 250 were eligible for and referred to ESWT treatment. We screened these 250 patients, out of which 120 patients were eligible to participate in the study. After explaining the purpose of the study to these patients, 75 agreed to participate and were recruited into this prospective self-controlled study to evaluate the effectiveness of ESWT among CP/CPPS patients. The study was conducted during the period January 2017-June 2019 at Department of Urology at our institution, the biggest tertiary care facility in Doha, Qatar. The Institutional Review Board at our institution provided ethical approval for the study (IRB# 16264/16).

### Inclusion and exclusion criteria

Inclusion criteria were adult men>18 years of age, complaining of pelvic discomfort, defined as groin, genitals, bladder, perineal and lower abdominal pain for ≥3 months, with no urological abnormalities, and with NIH-CPSI total score ≥15, negative urine culture, failure of pharmacological and/or non-pharmacological therapies, including alpha blockers, antibiotics, antidepressants, anticholinergics, neuroleptics, phytotherapy, physical therapy and mental health therapy to alleviate the symptoms , as well as patient ability to understand, communicate and comply with the study.

Patients were excluded if diagnosed or recently suffered from chronic urethritis, urinary stones, bacterial or inflammatory CP/CPPS, seminal vesiculitis, urological cancers, urethral strictures, neurogenic bladder dysfunction, or intake of antimicrobial, anti-inflammatory medications, anti neuropathic agents or analgesics within four weeks prior to study enrolment. In addition, patients with documented history of prostatic intraepithelial neoplasia on biopsy, serum prostate-specific antigen levels >4 ng/ml, history of previous or recent spermatic cord denervation, prostate surgery or radiotherapy, acute urinary retention, and any history of drug/narcotic abuse were also excluded.

### Treatment protocol

Eligible patients were provided with ample information about the aim and purpose of the study, its steps, their role, and the period of involvement. Those who agreed to be included in the study received additional information about the treatment sessions, duration of each session, patients’ position, and any anticipated side effects. A final contraindication check list was conducted to ensure that all enrolled patients met the inclusion criteria. No additional pharmacotherapy was prescribed nor allowed during the treatment sessions and follow-up period.

Patients received a total of four treatment sessions, one week apart using the Duolith SD1 Ultra Device (Storz Medical company, Tuttlingen, Switzerland). Each session lasted approximately 22 minutes, and the intervention was in the lithotomy position. The probe was firmly attached to the skin, after the application of ultrasound gel to ensure good wave transmission. In line with others [10,12,14], treatment comprised 3000 shock waves per session at a rate of 150 impulses per minute. Repositioning of the probe was undertaken with every 500 impulses delivered. Energy levels were started at 0.1 and increased up to 2.5 mJ/m^2^ with a 3 Hz frequency.

### Evaluation of outcomes

The NIH-CPSI Questionnaire was used for evaluation of the outcomes (Box 1) [2]. It was self-completed by all patients on four occasions: before starting the first treatment session; after completing the four ESWT sessions (week 0 after completion of treatment); and then at 12 and 26 weeks after completion of treatment. Additional data were retrieved from the hospital’s patient electronic records database: these included age and other demographic data, medical/surgical history, previous treatment history for CPPS and CP, and other clinical information.

**Box 1.** Items of the NIH-CPSI questionnaire for the diagnosis of CP/CPPS

Pain or Discomfort
1. In the last week, have you experienced any pain or discomfort in the following areas? a. Area between rectum and testicles (perineum) b. Testicles c. Tip of the penis (not related to urination) d. Below your waist, in your pubic or bladder area
2. In the last week have you experienced: a. Pain or burning during urination? b. Pain or discomfort during or after sexual climax (ejaculation)?
3. How often have you had pain or discomfort in any of these areas over the last week? 0 Never 1 Rarely 2 Sometimes 3 Often 4 Usually 5 Always
4. Which number best describes your AVERAGE pain or discomfort on the days that you had it, over the last week? **NO PAIN** 0 1 2 3 4 5 6 7 8 9 10 **PAIN AS BAD AS YOU CAN IMAGINE**
**Urination**
5. How often have you had the sensation of not emptying your bladder completely after you finished urinating, over the last week? 0 Not at all 1 Less than 1 time in 5 2 Less than half the time 3 About half the time 4 More than half the time 5 Almost always
6. How often have you had to urinate again less than two hours after you finished urinating, over the last week? 0 Not at all 1 Less than 1 time in 5 2 Less than half the time 3 About half the time 4 More than half the time 5 Almost always
**Impact of symptoms**
7. How much have your symptoms kept you from doing the kind of things you would usually do, over the last week? 0 None 1 Only a little 2 Some 3 A lot
8. How much did you think about your symptoms, over the last week? 0 None 1 Only a little 2 Some 3 A lot
**Quality of life**
9. If you were to spend the rest of your life with symptoms just the way they have been during the last week, how would you feel about that? 0 Delighted 1 Pleased 2 Mostly satisfied 3 Mixed 4 Mostly dissatisfied 5 Unhappy 6 Terrible

NIH-CPSI [2]

## Literature review

A formal literature review was performed using PubMed and MEDLINE databases. The search terms ‘ESWT’ or ‘external shock wave therapy’ or ‘CPPS’ or ‘chronic prostatitis’ or ‘chronic pelvic pain syndrome’ were used to identify articles that had these terms in the title, abstract or keywords. The inclusion criteria were English language studies reporting male adult patients presenting with CP/CPPS. Search results were reviewed for relevance, and we purposefully selected seven high-quality studies that illustrated the variability in practice and evaluation of effectiveness of ESWT.

## Statistical analysis

Descriptive statistics summarized the demographic and clinical characteristics. Paired sample t-tests compared the pain, urinary symptoms, quality of life and total NIH-CPSI pretreatment scores with values at 0, 12 and 26 weeks after completion of ESWT. Results were presented as percentage frequency (%) of patients with changes in NIH-CPSI scores; and as mean ± standard deviation of NIH-CPSI scores. All statistical analyses were completed using statistical packages SPSS 19.0, and *P* value <0.05 was considered as statistically significant.

## Results

### General characteristics of the sample

The sample’s mean age was 37.9 ± 8.6 years (range 18–60 years), with 3 years median duration of symptoms (range = 0.1–20 years). A total of 58 patients (77.3%) were married. Seven patients were diabetic (9.3%), 9 were hypertensive (12%), and 2 had cardiac conditions (2.7%). Before and during the ESWT treatment cycles, these 7 diabetic patients had not received Carbamazepine, Gabapentin or any other medications used to treat diabetic neuropathy. A total of 21 patients complained of erectile dysfunction (28%), 25 were smokers (33.3%), while 8 were ex-smokers (10.7%). No side effects were reported after the treatment sessions (e.g. mild/moderate pain, swelling, bruising/discoloration, numbness/tingling in the applied area).

### Follow up

**Figure 1.**
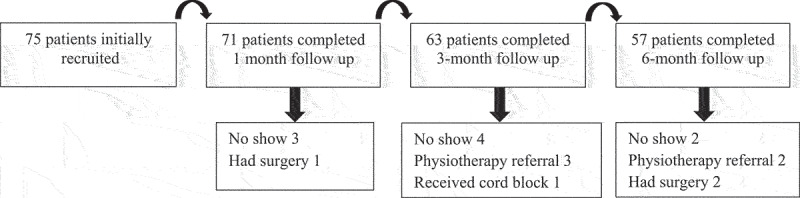
Patient flowchart.

Figure 1 shows the patient flowchart. Starting with 75 patients, 57 patients completed 6-month follow up. During the follow up period, 9 patients did not show up for the sessions at various stages of the treatment schedule, 5 preferred to be referred to physiotherapy after starting ESWT, one patient received cord block, and 3 underwent microsurgical spermatic cord denervation and hence were excluded from the analysis.

### Chronic prostatitis symptom index

Table 1 shows that regarding the NIH-CPSI scores, compared to pre-treatment scores, significant improvements were observed at week 0 post-treatment. In terms of patients’ response to treatment by NIH-CPSI domains, improvements were evident in the pain (96% of patients), urinary symptoms (64% of patients), and quality of life (74.8% of patients) domains; and 97.3% of patients exhibited overall improvements across all four NIH – CPSI domains (total score). As for the mean NIH-CPSI scores, significant improvements were also observed in the pain (5.2 ± 3.5 points), urinary symptoms (1.1 ± 2.1 points), and quality-of-life (2.8 ± 2.4 points) domains, as well as total score (9.2 ± 5.7 points).

**Table 1.** Differences in NIH-CPSI scores between pre-treatment and post-treatment week 0, 12 and 26 values.

Domain	Pre-Treatment value vs			P^*a*^	P^*b*^	P^*c*^
	PostT week 0	PostT week 12	PostT week 26			
	*n* = 71	*n* = 63	*n* = 57			
Pain	5.2 ± 3.4	4.55 ± 4.60	4.92 ± 4.69	<0.00I	<0.00I	<0.00I
Urinary symptoms	1.19 ± 2.18	0.76 ± 2.48	0.75 ± 2.96	<0.00I	0.018	0.060
Quality of life	2.88 ± 2.46	2.85 ± 2.78	2.50 ± 3.00	<0.00I	<0.00I	<0.00I
Total	9.26 ± 5.77	8.07 ± 7.56	8.29 ± 7.70	<0.00I	<0.00I	<0.00I

Based on pairwise analysis; Cell values represent pre-treatment value minus the value for the given post-treatment time point, positive values indicate an improvement; PostT; post- treatment; ^*a*^Pre-Treatment vs PostT week 0; ^*b*^Pre-Treatment vs PostT week 12; ^*c*^Pre-Treatment vs PostT week 26.

Patients’ NIH-CPSI scores exhibited significant improvements at 12 weeks after completing the ESWT compared to pre-treatment values. In terms of patients’ response to treatment by NIH-CPSI domains, improvements were evident in the pain (76.1% of patients), urinary symptoms (53.7% of patients), and quality of life (73% of patients) domains; and 80.9% of patients exhibited overall improvements across all four NIH – CPSI domains (total score). As for the mean NIH-CPSI scores, significant improvements were also observed in the pain (4.5 ± 4.6 points), quality-of-life (2.8 ± 2.7 points) domains, and total score (8 ± 7.5 points), while urinary symptoms (0.7 ± 2.4 points) improved but was not statistically significant.

At 26 weeks after completing ESWT sessions, 57 patients were still present in the study. Compared to pre-treatment scores, the NIH-CPSI scores showed significant improvements at 26 weeks post-treatment. In terms of patients’ response to treatment by NIH-CPSI domains, improvements were evident in the pain (78.9% of patients), urinary symptoms (61.4% of patients), and quality of life (75.4% of patients) domains; and 82.4% patients exhibited overall improvements across all four NIH – CPSI domains (total score). As for the mean NIH-CPSI scores, significant improvements were also observed in the pain (4.9 ± 4.6 points), quality-of-life (2.5 ± 3 points), domains, and total score (8.2 ± 7.7 points), while urinary symptoms (0.7 ± 2.9 points) improved but was not statistically significant.

## Discussion

ESWT is an emerging and promising CP/CPPS treatment modality [17]. Most studies of ESWT effectiveness among CP/CPPS patients had short term follow up of 8–12 weeks post treatment [10,11,15]. Long-term studies of the effectiveness of ESWT (6–12 months) had small samples of up to 31 patients [12–14]. The longest follow up evaluated 31 CP/CPPS patients who received ESWT after failure of combined oral alpha-blockers, antibiotics and anti-inflammatory therapy [14], and reported improvement in 26 patients (83.9%) that was maintained at 12 months [14]. The present study is one of the largest single arm studies of the longer term ESWT effectivness and safety, comprising 75 CP/CPPS patients diagnosed by clinical features and pre-treatment NIH-CPSI score, and followed up for 26 weeks after completion of treatment.

In connection with maximum improvement, in the current study, maximum improvement in overall NIH-CPSI score was directly after the last ESWT session (week 0 post-treatment), where 97.3% of patients improved (9.2 ± 5.7 points mean decrease from overall pre-treatment score), and improvement was significant across the three NIH-CPSI domains of pain, urinary symptoms and quality of life. Such improvement of symptoms immediately after treatment completion is consistent with previous reports [13,16], although it contrasts with other research, where maximum overall NIH-CPSI improvement was at later timepoints, namely 4, 8 or 12 weeks after completion of ESWT treatment [10,11,14,15]. While it is difficult to speculate the reasons behind such reported descripancies in the time of maximum improvement, it could be due to the different schemes that various studies employed. For instance, the number of treatment sessions (e.g. 4 vs 12 sessions) [10,13], time of first measurement during follow up (e.g. weeks 0 vs 1, vs 2 vs 8) [11,13,14,16], or the use of combination therapy [13,14] vs monotherapy [10,11,16] could influence the time of maximum improvement. Hence, some standardization/consistency across these aspects would facilitate better head-to-head comparisons across studies.

As for the durability of ESWT’s overall beneficial effects, others found that its effect was not sustained on the longer term, as there were no significant differences between the overall NIH-CPSI pre-treatment score and the final score 24 weeks after treatment completion [12]. We observed sustained effects at 26 weeks after treatment completion, where 82.4% of our patients maintained a mean 8.2 ± 7.7 points improvement from their pre- treatment overall NIH-CPSI (*P* < 0.05). However, this durable improvement was statistically significant for the pain and quality of life domains, but not for the urinary symptoms.

Certainly, compared to pre-treatment levels, urinary symptoms showed intial statistically significant improvement at week 0 after treatment completion that were not sustained on the longer term (at 12 and 26 weeks). Our observed short-term ESWT effect on urinary symptoms concurs with others, where urinary symptom scores, urine flowmetry and postvoiding residual exhibited temporary early improvement after ESWT that were not statistically maintained on longer follow up [10,12,13]. We are unable to speculate the reason behind such ‘waning’ of ESWT’s effect on voiding symptoms. An important point here is the difference between statistical significance vs clinical significance. We found a significant 1.19-point improvement at week 0 compared to pre-treatment level. Nevertheless, at weeks 12 and 26 weeks the score did not regress to its baseline pre-treatment levels, but rather, still showed improvements of 0.75/0.76 points (on a 5-point scale) which despite their statistical insignificance, in our view, represent clinically significant improvements that would definitely reflect in better quality of life of patients, particularly given the number of individuals suffering CP/CPPS. Further research would benefit to undertake evaluations of the natural history of the urinary symptoms among CP/CPPS patients, as well as longer term objective measurement of ESWT’s effects on urine flow and urodynamics.

In terms of mode of action, several theories have been proposed as to ESWT’s theurapetic effects on tissues [9]. For instance, ESWT stimulates the neovascularization process together with hyperstimulation of nociceptors resulting in the modification of nerve impulse flow [14], and also reduces muscle tone through the stimulation of nitric oxide (NO) synthesis [9]. NO stimulation occurs indirectley through enzymatic stimulation, or directly in response to the mechanical kinetics of the shock waves to tissue proteins [18]. In addition, it is suggested that NO plays an important role in ESWT’s anti-inflammatory effect [18]. Further, ESWT was found to have a regenerating role for the neuronal NO synthase-positive nerves, endothelium and smooth muscles of erectile tissue in rats, probably by recruiting mesenchymal stem cells [19].

Some studies compared the effects of triple therapy alone (oral alpha-blockers + antibiotics + anti-inflammatory agent) vs combined with 12-week ESWT sessions among CP/CPPS patients [13]. This study found that only the combined therapy group (but not the triple therapy group) showed significant improvement in the post-void residual and Qmax values [13]. On the other hand, although both study arms had improvements in all NIH-CPSI domains, the combined therapy exhibited significantly more improvement that was also sustained at 24 weeks after treatment completion compared to triple therapy [13]. Such findings suggest that ESWT *perse* exerts unique additional benefical effects on top of those of the triple therapy.

As for complications, throughout the current study, we observed no complications, and ESWT was well tolerated and accepted by patients. The 18 patients that dropped out of the present study were not because of complications or lack of tolerance to ESWT. Rather, these patients did not adhere to follow up visits as they became interested in starting physiotherapy or trying other treatment modalities. Our zero complication rate and good tolerance support previous reports confirmig ESWT’s safety and good tolerability as CP/CPPS management strategy [10,17].

A point to note is that our review suggested (Table 2) a wide varaiability across studies in terms of : 1) actual practice/application treatment protocols e.g. number of ESWT sessions, duration of treatment, characteristics of the short wave therapy in terms of device type, dose, total energy flow density, and duration of application per session; and 2) research methodology used to evaluate effectiveness e.g. duration of follow up (e.g. 8–52 weeks), sample size (e.g. 14–91 patients), presences of control group (e.g. no controls, sham controls, or controls receieving 30 triple therapy or combination therapy), evaluation time points, evaluation tool (e.g. Health-Chronic Prostatitis Symptom Index, International Prostate Symptom Score, International Index of Erectile Function, visual analog scale). Such variations reflect a lack of general consensus regarding the the application of ESWT as well as evaluation of its benefits/side effects.

**Table 2.** Extracorporeal shock wave therapy for chronic prostatitis/chronic pelvic pain syndrome: selected examples to illustrate variability*.

Author	Sample size/s	Control	Number of ESWT Sessions	TT period (wk)	SW characteristics: Device/Dose/Duration	FU (wk)	Evaluation time points (wk)/Tool^*a*^	Results	Maximum improvement
Current study	75 ESWT	—	4	4	Duolith 3000 SW, 0.1–0.25 mJ/mm^2^, 3 Hz 150 p/m, 20 min	26	PreT; then at 0, 12, 26 wk PostT/	Improvement in all 3 domains; improvement maintained except for urinary symptoms	Week 0 PostT; improved 9.2 ± 5.7 points from PreT
Moayednia 2014 [12]	19 ESWT	19 controls (sham)	4	4	DUOLITH SD1, Storz Medical, Tagerwilen, Switzer- ¨ land, 3000, 0.25 mJ/mm^2^ increasing by 0.05 mJ weekly to 0.45 mJ	24	16, 20, 24 wk PostT	No statistical difference between PreT vs 24 wk	No statistically significant improvement
Pajovic 2016^*b*^ [13]	30 combined (ESWT + triple therapy ^*g*^)	30 triple therapy ^*g*^	12	12	Lubisone, KM-2000 S, K1 Med Co. Ltd., Seoul, Korea. 3000 pulses, 0.25 mJ/mm^2^, 3 Hz, 12 min	24	PreT; then at 0, 12, 24 wk PostT	Both groups significantly improved, but combined TT improved more	Week 0 PostT
El Edwan 2017^*c*^ [16]	41 ESWT	—	4	4	E-S.W.T Roland, Pagani, Italy, 2500 pulses at one Bar pressure, maximum total energy flow density 0.25 mJ/mm^2^, 3 Hz, 13 min	52	PreT; then 2, 24, 52 wk PostT	All parameters^*c*^ significantly improved; effect maintained 52 weeks.	Wk 2 PostT
Salama 2018 [11]	20 ESWT	20 controls (sham)	8	4	Shockmaster Mp200, 3000, 12 Hz, 3–5 bar, 0.38 mJ/mm^2^	8	PreT; then then during TT at wk at 1, 4; then at 8 wk PostT	Significant improvement at 8 wk PostT across all domains	Wk 8 PostT
GUU 2019^*d*^ [14]	31 ESWT + medical treatment^*h*^	—	4	4	Duolith, Storz Medical, Tagerwilen, Switzer- ¨ land, 3000, 0.25 mJ/mm^2^, 4 Hz, 240 shocks/min, 6 different anatomical sites, 500 impulses each,	52	PreT; then at 1, 4, 12 wk PostT	Significant improvement except in patients with psychiatric disorders	Wk 12 PostT
Jin 2022^*e*^ [15]	91 ESWT	—	4	4	P100 Wikkon PT, 3 Hz, 0.2–0.3 mJ/mm, left and right perineal areas 1000 each	12	PreT; then during TT at wk 1, 2, 3,4; then at 1, 4, 12 wk PostT	Improvement across all domains, erectile function scores were not statistically significant	Wk 12 PostT
Zimmerman 2008^*f*^ [10] 2 studies	14 ESWT	—	6	2	Minilith SL1, Storz Medical, Tägerwilen, Switzerland, 2000 impulses, positive energy flow 0.11 mJ/mm^2^, 3 Hz, 12 min	12	PreT; then at 1, 4, 12 wk PostT	Pain and QoL significant improved, no statistical significance for voiding conditions	Wk 4 PostT
	20 ESWT	—	4	4	Duolith SD1, Storz Medical, Tagerwilen, Switzer- ¨ land,3000 impulses, total energy flow density 0.25 mJ/mm^2^, 3 Hz, 17 min				

*Variability in terms of device, dose/duration of shock waves, number of sessions, or tools employed to evaluate benefits; ESWT: extracorporeal shock wave therapy; TT: treatment; FU: follow up; wk: weeks; PostT: post-treatment; min: minutes; SW: shock wave; mJ: millijoules; PreT: pre-treatment; QoL: quality of life; ^a^All studies evaluated using National Institutes of Health -Chronic Prostatitis Symptom Index (NIH-CPSI) that include the domains of pain, quality of life, urinary symptoms; in addition, ^b^urine flowmetry and postvoiding residual; ^c^the International Prostate Symptom Score (IPSS), American Urological Association Quality of Life Due to Urinary Symptoms (AUA QOL_US) and International Index of Erectile Function (IIEF); ^d^visual analog scale, 5-item version of the International Index of Erectile Function and International Prostate Symptom Score ; ^e^IPSS, IIEF-5, Visual Analogue Scale (VAS); ^f^IPSS, VAS; ^g^ triple therapy (oral alpha-blockers + antibiotics + anti-inflammatory agent); ^h^Combined TTT to patients who failed response to medical TTT alone.

This study had limitations. The presence of a separate control group could have generated more concluisve evidence. In addition, evaluation of urinary symptoms was via self-reported NIH-CPSI score; objective urodynamic tests would have been useful to complement such subjective reporting. Increasing the follow up period would have strenghtened the reported effectivness findings of ESWT as a long term solution in pelvic pain management. Notwithstanding, the current study is one of the largest single arm studies evaluating ESWT’s effect on CP/CPPS symptoms for one of the longest follow up periods reported. The study sample is representative of the whole of Qatar, and not a single centre, as our facilitiy is the only tertiary care centre for managment of such patients in Qatar. Future research would benefit to assess the most effective ESWT therapy protocol, comparing e.g. different device settings, placement of the ESWT focus in relation to the points of maximal tenderness, as well as the frequency and spacing of treatment sessions.

## Conclusion

For CP/CPPS patients, ESWT is safe, accepted and well tolerated, with early relief of symptoms, starting directly after completion of treatment and effectively maintained through the long term for up to 26 weeks, particularly for the pain and quality of life domains. Urinary symptoms are intially improved but not statistically sustained on the longer term. Further evaluation of the treatment protocol/s that are most effective would be beneficial.
